# Tomatidine provides mitophagy‐independent neuroprotection after ischemic injury

**DOI:** 10.1002/2211-5463.13265

**Published:** 2021-08-23

**Authors:** Yu‐ting Wang, Li‐na Zhang, Xiao‐cui Lyu, Yue Li, Anil Ahsan, Zikai Feng, Xiangnan Zhang

**Affiliations:** ^1^ Department of Anesthesiology Sir Run Run Shaw Hospital School of Medicine Zhejiang University Hangzhou China; ^2^ Institute of Pharmacology and Toxicology Key Laboratory of Medical Neurobiology of the Ministry of Health of China College of Pharmaceutical Sciences Zhejiang University Hangzhou China

**Keywords:** cerebral ischemia, tomatidine, autophagy, mitophagy

## Abstract

Cerebral ischemia is one of the leading causes of human mortality and disability worldwide. The treatment of cerebral ischemia is refractory due to its short therapeutic window and lack of effective clinical drugs. Mitophagy, the autophagic elimination of damaged mitochondria, attenuates neuronal injury in cerebral ischemia, indicating the potential of mitophagy inducers as therapies for cerebral ischemia. We previously determined that, by enhancing autophagy flux, the steroidal alkaloid tomatidine can function as a neuroprotective agent against ischemic injury. However, its effects on mitophagy remain unknown. For this purpose, neuroblastoma cell lines Neuro‐2a and SH‐SY5Y were subjected to ischemic injury induced by oxygen–glucose deprivation/reperfusion (OGD/R) and then treated with tomatidine. OGD/R induced a general decrease of cellular contents, and this study revealed that tomatidine had no impact on mitophagy. In addition, tomatidine did not affect mitochondrial contents, including translocase of outer mitochondrial membrane 20 and voltage‐dependent anion channel 1, in either OGD/R‐treated or intact SH‐SY5H cells. Our results indicate that tomatidine exhibits its neuroprotective effects by enhancing autophagy, but in a potentially mitophagy‐independent manner, and provide insights for further investigation into its mechanism(s) and potential therapeutic use against cerebral ischemia.

## Graphical abstract

The steroidal alkaloid, tomatidine, has been shown to treat cerebral ischemia by enhancing autophagy, but its effect on mitophagy is still unknown. Our work indicates that the neuroprotective role of tomatidine is independent of mitophagy. Further research should be performed to determine how autophagy contributes to the neuroprotective effects of tomatidine in ischemic neurons.

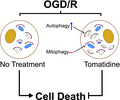

**Abbreviations**
CQ: chloroquine
DMEM: Dulbecco's modified Eagle’s medium
LDH: lactate dehydrogenase
N2a: Neuro‐2a
OGD: oxygen–glucose deprivation
OGD/R: oxygen–glucose deprivation/reperfusion
RIPA: radioimmunoprecipitation assay
SDC: sodium deoxycholate
SEM: standard error of mean
TOM20: translocase of outer mitochondrial membrane 20
VDAC1: voltage-dependent anion channel 1

Cerebral ischemia represents one of the refractory diseases worldwide because it causes mortality and permanent adult disability [1], while the underlying pathological mechanisms are not fully elucidated. Only until recent years has autophagy, the intracellular catabolic process that delivers cytosol and organelles to lysosomes for macromolecule turnover and recycling [2], drawn increasing attention in the context of cerebral ischemia, as well as a number of neurodegenerative disorders such as Parkinson’s disease [3, 4]. For cerebral ischemia, mounting evidence indicated that autophagy is activated in ischemic neurons and protects against neuronal injury in multiple cerebral ischemic models [5, 6, 7]. By eliminating damaged mitochondria (termed as mitophagy), autophagy prevents mitochondria‐dependent apoptosis and attenuates ischemia–reperfusion‐induced brain injury [3, 8]. Alternatively, reinforced mitophagy exhibited additional neuroprotection against ischemic injury [9, 10, 11, 12, 13]. These findings suggest the therapeutic potential of mitophagy activation against cerebral ischemic injury.

Emerging studies indicated that tomatidine, a steroidal alkaloid abundant in the *Solanaceae* family, could activate autophagy in varying species [14, 15]. Known as an anti‐inflammatory agent by blocking nuclear factor of kappa light polypeptide gene enhancer in B cells and c‐jun N‐terminal kinase signaling [16], tomatidine also exhibits immunostimulatory and cardioprotective effects and the ability to prevent muscle atrophy [17, 18]. To further investigate its therapeutic potential against cerebral ischemia, we previously reported tomatidine‐induced neuroprotection against ischemic injury by enhancing autophagic flux in Neuro‐2a (N2a) cells [19]. This was suggested to be achieved rather by autophagosome generation, but instead mainly through facilitating lysosomal degradation via activation of the transcription factor EB [19]. Even though it has the ability to promote lysosomal function, the effects of tomatidine on mitophagy were not determined in neuronal cells, although it was identified that tomatidine induces mitophagy in multiple other models [14, 15]. This study aims to identify whether mitophagy is involved in the neuroprotection of tomatidine in ischemic neuronal cells.

## Materials and methods

### Chemicals and reagents

Tomatidine (purity > 98.0%, HY‐N2149) was purchased from MedChemExpress (Shanghai, China). Penicillin, streptomycin, sodium deoxycholate (SDC), chloroquine (CQ) and poly‐L‐lysine were purchased from Sigma (Shanghai, China). DMSO and sodium chloride (NaCl) were purchased from Sinopharm (Shanghai, China). Stock solutions of tomatidine were freshly prepared at 100 mm in DMSO. High‐glucose and glucose‐free Dulbecco's modified Eagle’s medium (DMEM), FBS, N‐2 supplement (17502001) and B27 supplement (17504044) were purchased from Gibco (Shanghai, China). Penicillin–streptomycin was purchased from Solarbio (Beijing, China). WST‐1 assay kit was purchased from Beyotime (Shanghai, China). BCA protein assay kit was purchased from Yeasen (Shanghai, China). Carbonyl cyanide 3‐chlorophenylhydrazone was purchased from MCE (Shanghai, China). Tris was purchased from VWR (Shanghai, China). SDS was purchased from BioFroxx (Guangzhou, Guangdong, China). Triton X‐100 was purchased from Bomei (Hefei, Anhui, China). EDTA was purchased from Sangon (Shanghai, China). Sodium fluoride (NaF) was purchased from Amresco (Solon, OH, USA). Neurobasal Medium (21103049) was purchased from Invitrogen (Shanghai, China). Human kidney cDNA library (637204) and mito‐GFP (637204) were purchased from Clontech (Shanghai, China). JetPRIME transfection reagent was purchased from Polyplus Transfection (Shanghai, China). Protease inhibitor cocktail tablets (04693132001) were purchased from Roche (Shanghai, China). Translocase of outer mitochondrial membrane 20 (TOM20) Rabbit polyclonal antibody (A6774) was purchased from ABclonal (China). Voltage‐dependent anion channel 1 (VDAC1) Rabbit mAb (A19707) was purchased from ABclonal (China). GAPDH loading control (KC‐5G4) was purchased from KangChen (Shanghai, China). Peroxidase AffiniPure goat anti‐mouse (115‐035‐003) and goat anti‐rabbit (111‐035‐003) secondary antibodies were purchased from Jackson ImmunoResearch (West Grove, PA, USA).

### Cell culture

Human (SH‐SY5Y, CRL‐2266) and mouse (N2a, CCL‐131) neuroblastoma cells (ATCC, Shanghai, China) were routinely cultured in high‐glucose DMEM (supplemented with 10% FBS, 100 U·mL^−1^ penicillin and 0.1 mg·mL^−1^ streptomycin) at 37 °C in standard atmosphere (95% humidified air + 5% CO_2_). The cells were passaged when reaching ˜75% confluency.

### Oxygen–glucose deprivation/reperfusion

To mimic ischemia/reperfusion‐like conditions, an oxygen–glucose deprivation/reperfusion (OGD/R) model was used as previously reported with minor modifications [20]. In brief, seeded cells were rinsed with PBS and glucose‐free DMEM, placed in a sealed chamber (MIC‐101; Billups‐Rothenberg, Del Mar, CA, USA), loaded with oxygen‐free atmosphere (95% N_2_ + 5% CO_2_, 25 L·min^−1^, 6 min), followed by incubation (4 h, 37 °C) and refreshed with O_2_‐ and glucose‐free DMEM (prebalanced in an O_2_‐free chamber at 37 °C). Subsequently, cells were refreshed with high‐glucose DMEM with or without treatment in standard atmosphere. In contrast, cells refreshed with high‐glucose DMEM and incubated in standard atmosphere were used as an oxygen–glucose deprivation (OGD)‐negative control. See specific values for different assays detailed in the following subsections.

### Quantification of mitophagy level

As previously described [19], the human microtubule‐associated protein 1 light chain 3 beta cDNA was amplified from the human kidney cDNA library by PCR with the forward primer 5′‐CGC AGA TCT ATG CCG TCG GAG AAG ACC TTC‐3’ and the reverse primer 5′‐GCC GAA TTC CGC TTA CAC TGA CAA TTT CAT CCC G‐3′. The PCR product was then inserted into the *BglII* and *EcoRI* sites of mCherry‐C1 plasmid to construct the mCherry‐LC3 plasmid.

N2a cells were seeded (1.5 × 10^5^ cells per dish, 2 mL per dish) in glass‐bottom dishes (D35‐20‐0‐N; Cellvis) precoated with poly‐l‐lysine (0.1 mg·mL^−1^, 2 mL·per dish, 2 h) and left to adhere overnight. Subsequently, media were replaced with DMEM (2 mL) containing N‐2 supplement (2%, 6 h) for differentiation into neuron‐like cells, as well as mCherry‐LC3 and GFP‐LC3 plasmids (2 μg each, 6 h) for transfection, using a transfection kit according to the manufacturer’s instructions. After media removal, cells were further differentiated using N‐2 supplement (2% in DMEM, 2 mL·per dish, 18 h), followed by tomatidine pretreatment (0, 1, 3 and 10 μm, 1 h). After OGD as described earlier, cells were refreshed with tomatidine reperfusion (0, 1, 3 and 10 μm, 24 h).

After OGD/R, cells were observed under a confocal microscope [Leica TCS SP8, 63 × (NA‐1.4) oil‐immersion; Shanghai, China] in standard atmosphere, and Z‐stack images were acquired (resolution, 1024 × 1024 pixels; step size, 0.3–1 µm). The average area of mito‐GFP puncta and Mander’s overlap efficiency were measured and analyzed as described previously [21] using Image Pro‐Plus software (version 7.0; Media Cybernetics, Rockville, MD, USA). At least 50 cells were analyzed per condition, and data were standardized on the negative control from three independent assays.

### Quantification of cytotoxicity and cytoprotection

SH‐SY5Y cells were seeded (5 × 10^3^ cells per well, 100 μL·per well, 96‐well plate) and left to adhere overnight for WST‐1, lactate dehydrogenase (LDH) or protein assay. After OGD, as described earlier in this chapter, reperfusion was performed by replacing media with high‐glucose DMEM (100 μL·per well) containing tomatidine (0, 1, 3 and 10 μm, 24 h). To indicate viability, we used NAD(P)H level as a surrogate marker measured by supplementing WST‐1 reagent (10 μL·per well, 1 h, 37 °C, absorbance at 450 nm) according to the manufacturer’s instructions. To indicate cellular membrane integrity, we used extracellular LDH level as a surrogate marker using an LDH release assay kit. Culture media were transferred to a separate plate (80 μL per well, 96‐well plate) and supplemented with reagent mixture according to the manufacturer’s instructions (40 μL·per well, 1 h, room temperature, absorbance at 490 nm). After complete removal of media, cells were washed with PBS (100 μL) and permeabilized with radioimmunoprecipitation assay (RIPA) lysis buffer (20 mm Tris, pH 7.5, 150 mm NaCl, 0.5% SDC, 0.1% SDS, 1% Triton X‐100, 1 mm EDTA, 20 mm NaF; 50 μL, 10 min). Protein concentration of cell lysates (20 μL) was determined using a BCA protein assay kit according to the manufacturer’s instructions (140 μL per well, 30 min, 37 °C, absorbance at 570 nm). A standard curve of BSA (0–2 mg·mL^−1^) was used for protein quantification. OGD‐negative controls followed by tomatidine treatment at the maximal test concentration (10 μm, 24 h) was used to indicate inherent toxicity of tomatidine. Data were standardized on the OGD‐negative controls (100%) and expressed as mean ± standard error of mean (SEM) from at least three independent experiments with four parallel wells per experiment.

### Quantification of mitochondrial contents

SH‐SY5Y cells were seeded (1.5 × 10^5^ cells in 2 mL·per well, six‐well plate) and allowed to adhere overnight. The cells pretreated with CQ (15 μm, 2 mL per well, 4 h) before OGD were used as an autophagic flux measurement. After OGD as described earlier in this chapter, cells were refreshed with tomatidine reperfusion (0, 1, 3 and 10 μm, 6 h). After media removal, cells were washed with PBS (ice‐cold, pH 7.4, 1 mL) and stored in PBS (1 mL), collected using a cell scraper (Sarstedt, Shanghai, China). The cells were centrifuged (100 × ***g***, 4 min, 4 °C) and homogenized using RIPA lysis buffer (ice‐cold, 30–50 μL, 20 mm Tris–HCl, pH 7.4, 150 mm NaCl, 0.5% SDC, 0.1% SDS, 1% Triton‐X 100, 1 mm EDTA, 20 mm NaF, 2% protease inhibitor cocktail in PBS, 20 min, on ice). After centrifugation (12 000 × ***g***, 20 min, 4 °C), supernatant was collected and protein concentration was determined using BCA Protein Assay kit as instructed by the manufacturer. After adjusting concentration (2 mg·mL^−1^ in RIPA containing 1 × SDS loading buffer), proteins were denatured (5 min, 100 °C) and stored at −80 °C until used.

An aliquot of 20 µg protein samples was loaded on 12.5% SDS/PAGE gel, followed by blotting on nitrocellulose membrane (in transfer buffer containing 20% methanol) and blocking (5% milk in PBS, pH 7.4, 1 h, room temperature). After washing with PBST (0.16% Tween 20/PBS, 5 min, 3×), target proteins were exposed to rabbit anti‐TOM20 Ig (1 : 1000), rabbit anti‐VDAC1 Ig (1 : 1000) and rabbit anti‐β‐actin Ig (1 : 3000) (4 mL in PBST, overnight, 4 °C), respectively. After another three times washing with PBST, target proteins were probed with goat anti‐rabbit secondary antibodies (1 : 3000, 4 mL in PBST, 1 h, room temperature). Immunoblots were visualized using ECL detection reagents and an infrared imaging system (Odyssey 9120; LI‐COR Biosciences, Shanghai, China). Densitometric analysis on the immunoblots was performed using imagej software (Bethesda, MD, USA). The optical density of target proteins was normalized on that of β‐actin.

### Statistical analysis

All data were collected and analyzed in a blinded manner and expressed as mean ± SEM. graphpad Prism (version 8.2; San Diego, CA, USA) was used for one‐way ANOVA of multiple comparisons, and *P* < 0.05 was considered statistically significant.

## Results and Discussion

### Tomatidine protects SH‐SY5Y cells against ischemic injury without cytotoxicity

**Fig. 1:**
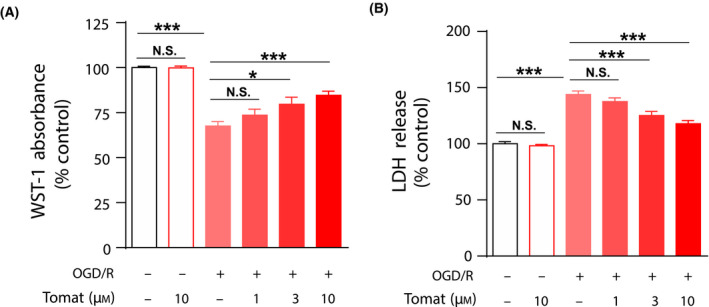
Tomatidine (Tomat) protects SH‐SY5Y cells against ischemic injury without cytotoxicity. After 4‐h OGD, SH‐SY5Y cells were reperfused (OGD/R) with 1, 3 or 10 µm tomatidine treatment for 6 h. Effects of OGD/R and tomatidine were depicted by (A) quantification of WST‐1 absorbance. (B) Quantification of LDH release. One‐way ANOVA was performed using graphpad Prism (version 8.2): *P < 0.05, ***P < 0.001 versus indicated group *n* = 12. Error bars, SEM. N.S., nonsignificant.

In this study, we aimed to deepen the understanding of tomatidine neuroprotective mechanism(s) against ischemic neuronal injury. First, we investigate the toxicity of tomatidine. In our test model, OGD/R significantly reduced the rate of cell proliferation, whereas tomatidine treatment had no effect. Moreover, as the dose of tomatidine (1, 3 and 10 µm) increased, the cell proliferation rate increased (Fig. 1A). Consistent with these results, we used LDH to detect cell viability, and OGD/R significantly reduced the rate of cell viability, whereas tomatidine treatment reverses that phenomenon (Fig. 1B).

### Tomatidine does not reinforce mitophagy in ischemic N2a cells

**Fig. 2:**
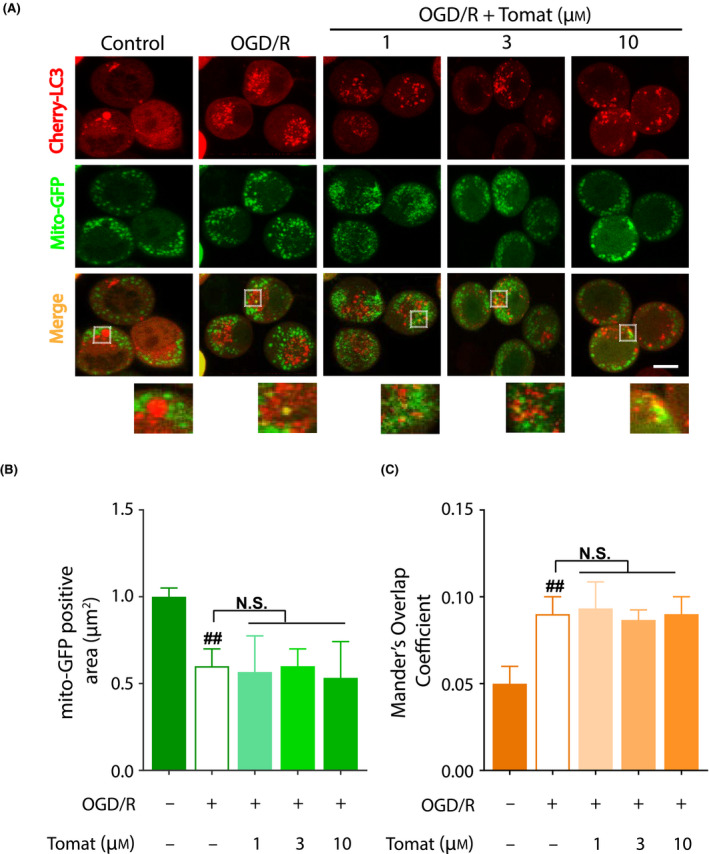
Tomatidine (Tomat) does not reinforce mitophagy in ischemic N2a cells. After 4‐h OGD, N2a cells were reperfused (OGD/R) with 1, 3 or 10 µm tomatidine treatment for 6 h. Mitochondria and LC3 puncta were visualized by mCherry‐LC3 and mito‐GFP, respectively. OGD/R significantly reduced mito‐GFP‐labeled mitochondria and increased the Mander’s overlap of mito‐GFP‐labeled mitochondria and mCherry‐LC3‐labeled LC3 puncta in N2a cells, whereas none of the parameters were reinforced by tomatidine. Effects of OGD/R and tomatidine were depicted by (A) representative fluorescent images captured by confocal microscopy, (B) quantification of mito‐GFP‐positive area and (C) Mander’s overlap coefficient of mito‐GFP‐ and mCherry‐LC3‐positive area in N2a cells. At least 50 cells were analyzed per condition, and data were standardized on the negative control from three independent assays. One‐way ANOVA was performed using graphpad Prism (version 8.2): ^##^ *P* < 0.01 versus control group. Scale bar: 20 μm. Error bars, SEM. N.S., nonsignificant.

Based on our previous report that tomatidine induces autophagy [19] and that tomatidine induces mitophagy in mammal cells and *Caenorhabditis elegans* [14], tomatidine was further assessed for its inducing potential of mitophagy. On the accumulation of mCherry‐LC3 in autophagosomes and mito‐GFP in mitochondria, autophagosomes appear as red puncta compared with mitochondria as green puncta. As such, the colocalization of autophagosomes and mitochondria appear as yellow puncta that is merged from the two fluorescences. Therefore, the number of yellow puncta indicates the level of mitophagy. In our test model, OGD/R significantly reduced the area of mito‐GFP‐labeled mitochondria in N2a cells (*P* < 0.01), which suggested mitophagy activation (Fig. 2A). Nevertheless, no significant differences of the area of mito‐GFP‐labeled mitochondria were observed between OGD/R‐treated and tomatidine‐treated groups (1, 3 and 10 µm) (Fig. 2B). Consistent with these results, OGD/R significantly increased Mander’s overlap coefficient of mito‐GFP‐labeled mitochondria and mCherry‐LC3‐labeled LC3 puncta in N2a cells (*P* < 0.01), suggesting mitophagy activation (Fig. 2C). Again, no significant differences of the Mander’s overlap coefficient was observed between OGD/R‐treated and tomatidine‐treated groups (1, 3 and 10 µm) (Fig. 2C).

### Tomatidine does not affect mitochondrial content of SH‐SY5Y cells

**Fig. 3:**
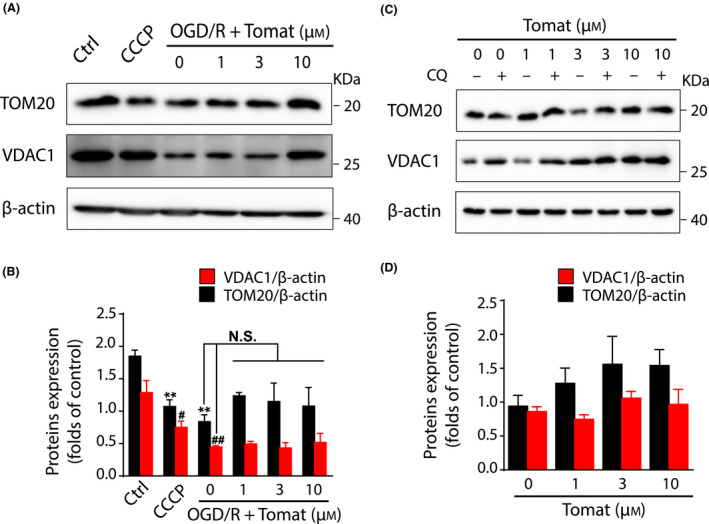
Tomatidine (Tomat) does not affect mitochondrial content of SH‐SY5Y cells. TOM20 and VDAC1 expression were determined by western blot and quantified in (A,B) OGD/R‐ or carbonyl cyanide 3‐chlorophenylhydrazone (CCCP)‐treated and (C,D) intact cells (*n* = 3). SH‐SY5Y cells were treated with 100 μm CCCP for 6 h. Ischemic cells were treated with 4‐h OGD after 6‐h reperfusion containing 0, 1, 3 and 10 μm tomatidine treatment. Autophagy in intact cells was inhibited by 10 μm CQ. One‐way ANOVA was performed using GraphPad Prism (version 8.2): **P < 0.01 versus TOM20 in control (Ctrl) group; ^#^ *P* < 0.05, ^##^ *P* < 0.01 versus VDAC1 in control group. Error bars, SEM. N.S., nonsignificant.

To further verify whether tomatidine affects mitochondrial contents, we further determined the expression of the widely used mitochondrial markers TOM20 and VDAC1 OGD/R‐treated and intact SH‐SY5Y cells by western blot. The oxidative phosphorylation uncoupler carbonyl cyanide 3‐chlorophenylhydrazone was used to induce mitophagy as a positive control. The lysosome inhibitor CQ was used to inhibit autophagy by altering lysosomal pH upon the diffusion into the lysosome and conversion into the diprotonated form [23]. The results showed that carbonyl cyanide 3‐chlorophenylhydrazone treatment and OGD/R significantly reduced TOM20 and VDAC1 expression, whereas neither of the two markers was further reduced by tomatidine at all test concentrations (Fig. 3A,B). Similarly, none of the test concentrations of tomatidine altered TOM20 and VDAC1 expression even in intact or CQ‐treated SH‐SY5Y cells (Fig. 3C,D). Other blots were included in Fig. S1.

## Conclusions

The development of effective therapeutics against cerebral ischemia has been challenging worldwide. As a novel therapeutic candidate, tomatidine is able to cross the blood–brain barrier identified with neuroprotection from ischemic injury in the context of neurodegenerative disorders such as Alzheimer’s disease [22, 23]. Studies using varying tissues and/or organs provide mounting evidence and indicate that tomatidine‐induced cytoprotection against ischemic injuries was associated with antioxidant, anti‐inflammatory and anticancer activities [16, 17, 22]. Furthermore, we reported that tomatidine induced autophagic flux against ischemic injury by activating lysosomal functions *in vitro* [19], suggesting the pharmacological basis of tomatidine and its use as a promising therapy against cerebral ischemia. Based on our knowledge that autophagy confers neuroprotection by promoting mitophagy [3], this study aimed to further characterize the ability of tomatidine to induce mitophagy using the same test model.

Although our test model successfully mimicked ischemia–reperfusion‐like conditions, to our surprise, tomatidine did not reinforce mitophagy in differentiated N2a cells, as supported by analysis of mitochondria alone and the colocalization of mitochondria and autophagosomes. Furthermore, by quantifying the most representative mitochondrial markers VDAC1 and TOMM20, our results suggested that tomatidine was also devoid of impact on mitochondrial contents in neither ischemic nor intact cells, despite inhibition of autophagy by CQ. These negative observations could be attributed by the high level of reactive oxygen species in our test model, which was also reported in ischemia–reperfusion‐treated neurons [9]. Alternatively, the effects of tomatidine on mitochondria could be exhibited in a delayed manner and specific timescale in neurons [24] that may not be detectable in the model used for this study, which will require further in‐depth investigations.

Taken together, this study suggests that mitochondria may not be associated with tomatidine‐conferred neuroprotection against ischemic injury and provided insights into deeper understanding of the mechanism underlying its neuroprotection. Although the causality of tomatidine on mitophagy remains to be elucidated in future investigations, tomatidine significantly induces neuroprotection against ischemic injury by enhancing autophagy. Together with the favorable bioactivities and safety as a natural product‐derived compound, tomatidine exhibits its promising therapeutic potential against cerebral ischemia.

## Conflict of interest

The authors declare no conflict of interest.

## Data accessibility

The data that support the findings of this study are available from the corresponding author (xiangnan_zhang@zju.edu.cn) upon reasonable request.

## Author contributions

Y‐tW: conceptualization, investigation and writing – original draft; L‐nZ: methodology, validation and data curation; X‐cL: methodology, validation and data curation; YL: investigation and writing; AA: formal analysis and visualization; ZF: writing – review and editing, and visualization; XZ: conceptualization, investigation, funding acquisition, resources and project supervision.

## Supporting information

**Fig**. **S1**. Tomatidine does not affect mitochondrial content of SH‐SY5Y cells. TOM20 and VDAC1 expression was determined by western blot carbonyl cyanide 3‐chlorophenylhydrazone treated (A,B) or OGD/R (C,D). SH‐SY5Y cells was treated with 100 μm carbonyl cyanide 3‐chlorophenylhydrazone for 6 h. Ischemic cells were treated with 4 h OGD following 6 h reperfusion containing 0, 1, 3 and 10 μm tomatidine (Tomat) treatment. Autophagy in intact cells was inhibited by 10 μm chloroquine (CQ).
