# Supplementary figures and images for: Tomatidine provides mitophagy‐independent neuroprotection after ischemic injury
Source: FEBS Open Bio. 2021 Aug 23;11(9):2647–54. doi: 10.1002/2211-5463.13265 (PMC8409302; doi:10.1002/2211-5463.13265)

**A**

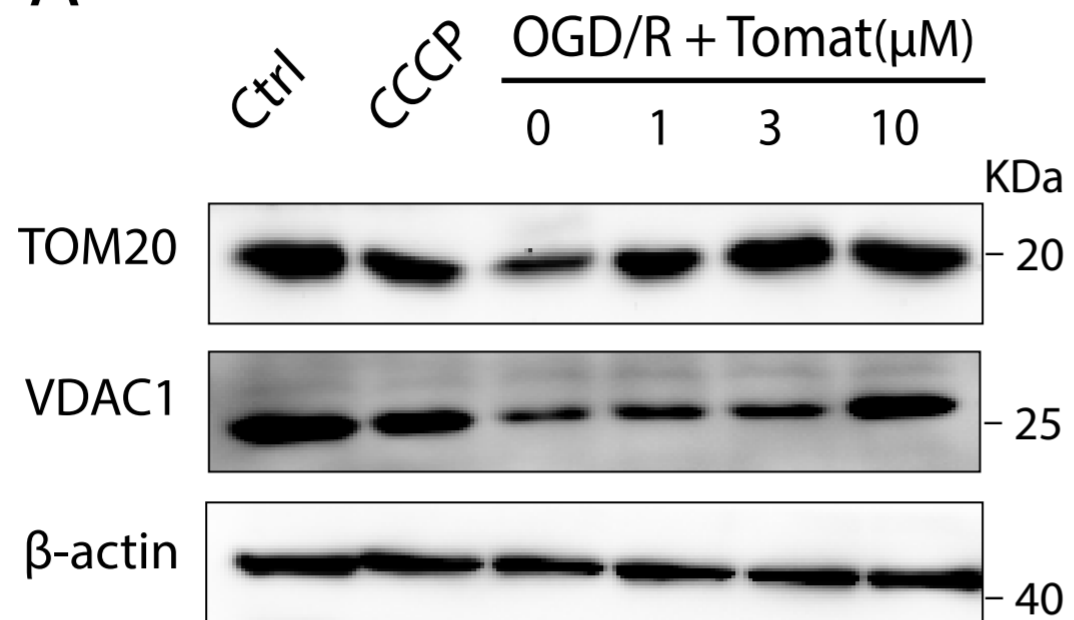

**B**

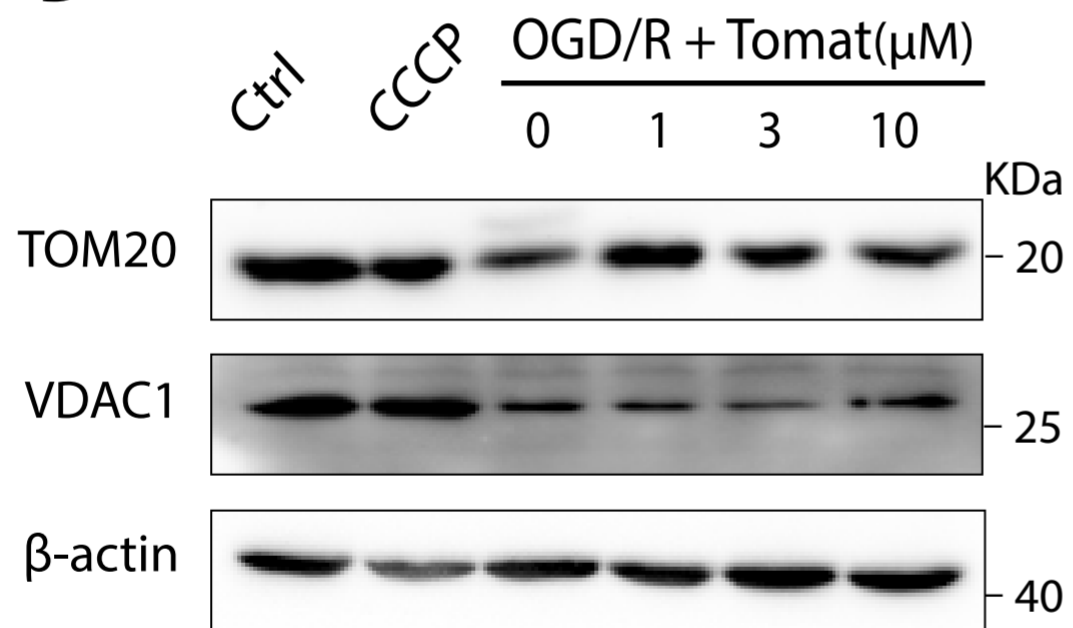

**C**

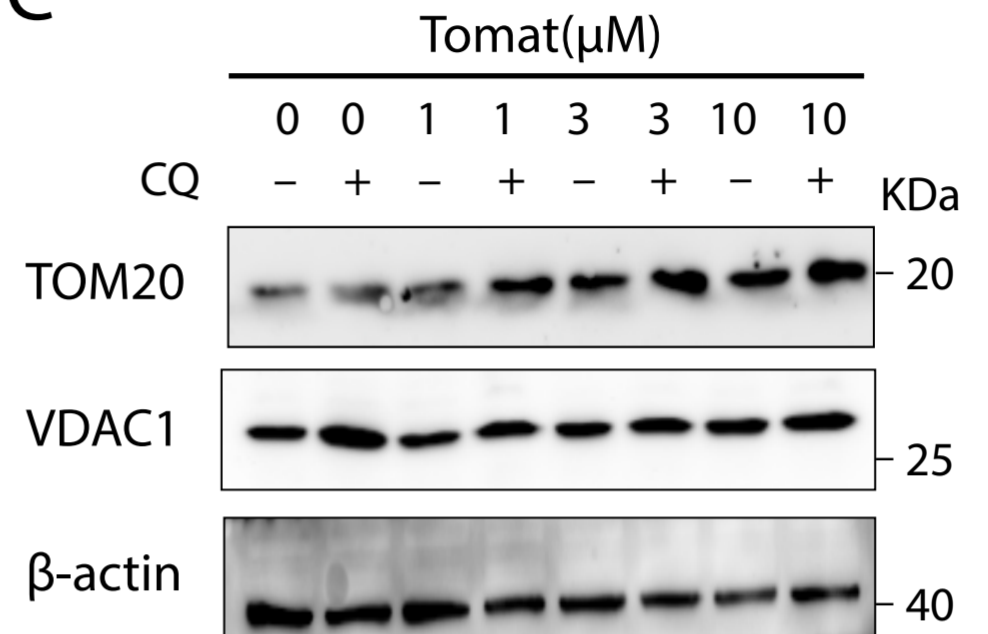

**D**

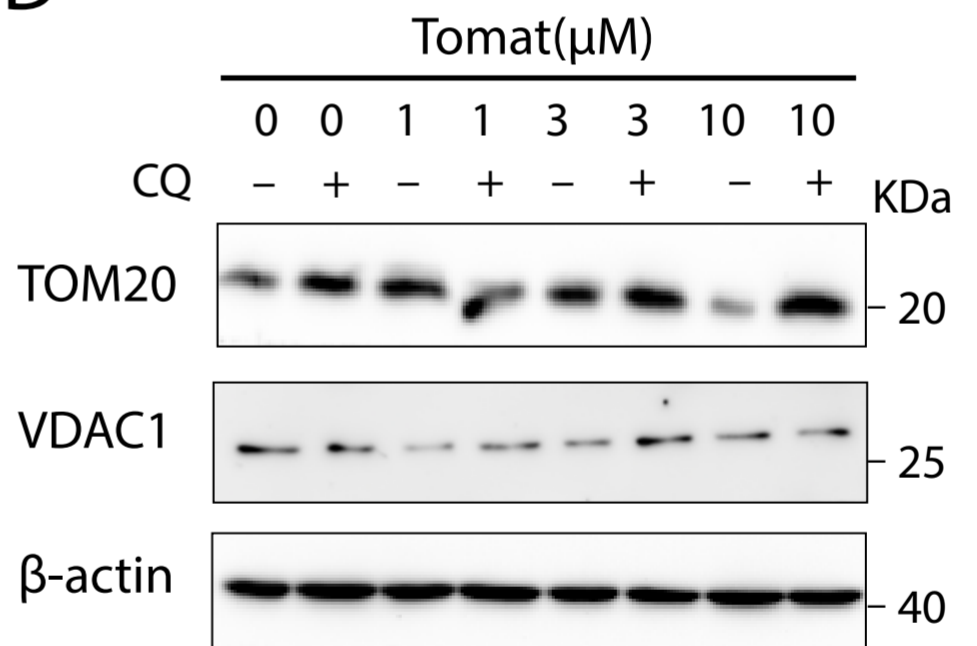

Supplement: Supplementary file 1 — Fig. S1. Tomatidine does not affect mitochondrial content of SH‐SY5Y cells. TOM20 and VDAC1 expression was determined by western blot carbonyl cyanide 3‐chlorophenylhydrazone treated (A,B) or OGD/R (C,D). SH‐SY5Y cells was treated with 100 μm carbonyl cyanide 3‐chlorophenylhydrazone for 6 h. Ischemic cells were treated with 4 h OGD following 6 h reperfusion containing 0, 1, 3 and 10 μm tomatidine (Tomat) treatment. Autophagy in intact cells was inhibited by 10 μm chloroquine (CQ). [file FEB4-11-2647-s001.pdf]
